# Dendrimers as Modulators of Brain Cells

**DOI:** 10.3390/molecules25194489

**Published:** 2020-09-30

**Authors:** Dusica Maysinger, Qiaochu Zhang, Ashok Kakkar

**Affiliations:** 1 Department of Pharmacology and Therapeutics, McGill University, 3655 Promenade Sir William Osler, Montreal, QC H3G 1Y6, Canada; qiaochu.zhang@mail.mcgill.ca; 2 Department of Chemistry, McGill University, 801 Sherbrooke St West, Montreal, QC H3A 0B8, Canada

**Keywords:** dendrimer, drug delivery, brain cells, nanomedicine, microglia, astrocytes, inflammation, glioblastoma

## Abstract

Nanostructured hyperbranched macromolecules have been extensively studied at the chemical, physical and morphological levels. The cellular structural and functional complexity of neural cells and their cross-talk have made it rather difficult to evaluate dendrimer effects in a mixed population of glial cells and neurons. Thus, we are at a relatively early stage of bench-to-bedside translation, and this is due mainly to the lack of data valuable for clinical investigations. It is only recently that techniques have become available that allow for analyses of biological processes inside the living cells, at the nanoscale, in real time. This review summarizes the essential properties of neural cells and dendrimers, and provides a cross-section of biological, pre-clinical and early clinical studies, where dendrimers were used as nanocarriers. It also highlights some examples of biological studies employing dendritic polyglycerol sulfates and their effects on glia and neurons. It is the aim of this review to encourage young scientists to advance mechanistic and technological approaches in dendrimer research so that these extremely versatile and attractive nanostructures gain even greater recognition in translational medicine.

## 1. Introduction

Despite tremendous advances in nano- and biotechnology, the products coming from these exciting areas of research are only beginning to be applied, validated and proven useful in clinics [1,2,3,4]. One of the reasons for their lag in solving numerous medical problems is not only our limited knowledge of complex human and animal pathologies, but also an inadequate sensitivity, specificity and sometimes toxicity of the nanotechnological constructs. We need new and better nanostructures that would satisfy the basic criteria to make them acceptable for diagnostic and therapeutic purposes in humans. Therefore, nanostructures have been developed to show increased bioavailability, prolonged circulating time, better stability, lower non-selective toxicity, and the ability to be functionalized. Among the building blocks for complex nanostructures, many scientists have resorted to nature, and have employed naturally occurring monomers and polymers, including chitosan and alginate, for applications in biology [5]. These natural polymers cannot be easily controlled in terms of composition and purity, and standardization protocols are still not available. On the other hand, synthetic polymers can be tailored through chemical procedures to generate imaginative new structures with different sizes, shapes and biological properties. Among the best-controlled nanostructures in a limited number of clinical trials are dendrimers [6]. The aim of this mini-review is to highlight the properties and outcomes from pre-clinical and clinical studies with dendrimers as nanocarriers or imaging agents. We present here examples of dendrimers with or without drugs incorporated in them and highlight their main advantages and limitations, mainly affecting the brain cells. Dendrimers are versatile nanostructures owing to their tenability of sizes, branch compositions and the ability to functionalize these branches. Some examples highlighted here also show responsiveness to endogenous stimuli such as changes in pH, concentrations of reactive oxygen species, temperature, and neurotransmitters (e.g., acetylcholine). First, we provide below a brief account of some basic features of neural cells and their communications, indicating the main problems, and, subsequently, some success in biomedical research describing the biological nanostructures and applications of dendrimers.

## 2. Neurons and Glial Cells in the Brain

### 2.1. Neurons

Homeostatic communications between the neural cells and neurovasculature in the brain are essential for the physiological functions of the central nervous system (CNS). The efficacy of these communications depends on the integrity of neurons and glia, synapses and the nanostructures called tunneling nanotubes (TNTs) [7,8,9]. The physical characteristics of TNTs can spread over a long-range, with lengths up to 200 μm and diameters from 0.5 to 0.7 μm, depending on how these structures are defined or identified. Recent studies by Di Polo’s group have shown that nanotube-like processes (named interpericyte tunneling nanotubes) connect pericytes on separate capillary systems, forming a functional network in mouse retina [10]. These nanotubes carry organelles such as mitochondria and signaling molecules, and also serve as conduits for Ca^2+^ waves, thus mediating the interpericyte communications. Communications between neural cells are triggered by numerous biochemical, electrical and mechanical stimuli. The intensity, duration and location of different stimuli determine the physiological and pathological functions within the brain structures. Communications between neurons and glia occur largely via synapses, nanostructures of about 20–40 nm, in one or more dimensions. Synapses are biological nanostructures which play essential roles in the CNS functions [11,12,13]. The neuronal cell body (soma) has a fine tubular process called an axon which conducts electrical impulses from the soma to the axon terminals (presynaptic boutons). Neuronal somas have multiple fine processes called dendrites, and they serve as the primary structures for the reception of synaptic contacts from other neurons and glia. Dendrites form the neurons typically receive information from 1000 to 10,000 synapses. Changes in signaling states control the synaptic strength, which is considered the basis of brain plasticity. Among the most important modulators of synaptic networks in the brain are diverse signaling proteins and ions, e.g., Ca^2+^. In the resting state, Ca^2+^ concentrations are often between 50 and 100 nM, but they increase 10–100-fold when neurons are activated. Ca^2+^ pulses regulate the structure and function of synapses and dendritic excitability [14,15,16,17]. The diffusion of signaling molecules is efficient at the micrometer scale and plays a critical role in signaling activities. A mathematical approach describing the balance between neurotransmitter diffusion and inactivation rate is described by Yasuda [18]. Similar studies employing various dendrimers that could form complexes with calcium would be of interest to measure if and how dendrimers could modulate the diffusion of signaling molecules and the extent of binding to calcium ions. Yasuda’s study gives calculated and experimental diffusion values for several signaling molecules and provides basic information for conducting similar studies in the presence of dendrimers. Longitudinal studies at the nanoscale related to biological processes in the brain structures were limited due to the inadequate resolution and accuracy at such a level. Today, we are able to follow the movements of some neurotransmitters and other neuroactive molecules. Such studies present an exciting new platform for dendrimer-signaling molecules’ interactions and biological outcomes at the single-cell level. Among the neurons, pyramidal neurons were often studied in neurodegeneration related to aging and genetic mutations. Our laboratories studied them in organotypic cultures in the presence of polyglycerol dendrimers.

**Figure 1:**
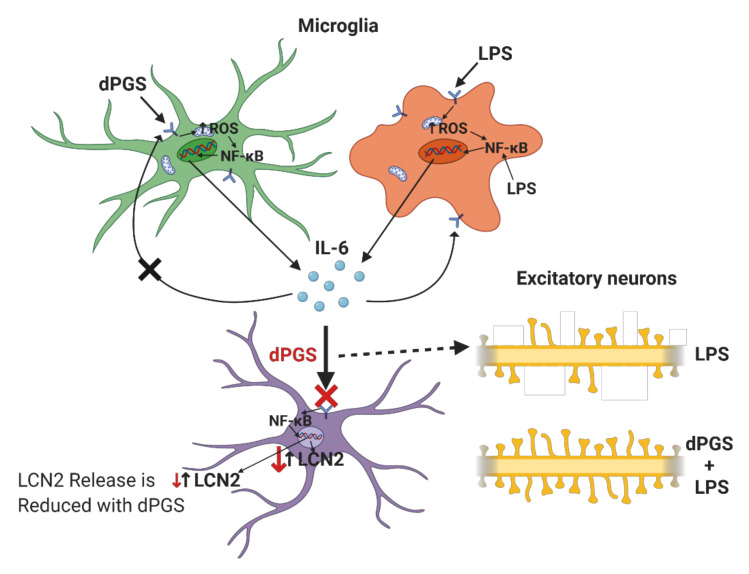
Differential anti-inflammatory actions of dendritic polyglycerol sulfate (dPGS) on neural cells in CNS: IL-6 = Interleukin-6; LPS = Lipopolysaccharide; LCN2 = Lipocalin-2; ROS = Reactive oxygen species; NF-κB = Nuclear factor kappa B.

Dendrites of pyramidal neurons in the hippocampus are decorated with dendritic spines, fine protrusions emanating from the dendritic surfaces. Dendritic spine morphologies include stubby, mushroom and thin spines, and their shapes determine synaptic strengths [19,20,21]. Dendritic spines have a small “head” and narrow “neck”, both of which are within the nanoscale dimensions. For example, the mature spine of the mushroom type has a head of 0.01–1 um^3^ and a neck from 100 nm to 1 um long and about 100 nm in diameter. Loss of synaptic spines results in impaired or lost neuronal functions. Thus, drugs and nanotherapeutics are designed and tested to prevent dendritic spine loss and preserve cerebral homeostasis. We have tested polyglycerol dendrimers with sulfate groups to prevent the loss of spines in models of endotoxemia caused by lipopolysaccharide (LPS) (Figure 1) [22]. Considering that the functions of neurons are dependent on glial cells in their environment, it is important to investigate the effects of dendrimers on astrocytes and microglia. The following sections briefly summarize the key features of these glial cells, and the reported studies on this subject are included in Table 1.

### 2.2. Astrocytes

Astrocytes represent the most prominent type of glial cell in the brain and the tumor microenvironment, which plays a pivotal role in nurturing and protecting neurons. Damage to these glial cells is detrimental to normal neuronal function [22,23,24,25,26]. Dysfunctional and reactive astrocytes contribute to the progression of many brain disorders and diseases, especially glioblastoma—hence, understanding the role of human astrocytic response to nanotherapy is essential [26,27,28,29,30]. However, data regarding human astrocytes and their interactions with dendrimers are sparse [22,31,32,33,34]. In contrast, extensive studies have been carried out with gold nanostructures including gold nanoclusters in rodent models, but these cannot be directly translated to the human cell, because of the differences between the morphology, genetic profile and responsiveness of human and rodent astrocytes. Our group has been particularly interested in the control of transcription factors in astrocytes exposed to nanostructured materials, including dendrimers. For example, an alarmin High Mobility Group Box 1 (HMGB1) is a transcription factor conserved and expressed in all nucleated animal cells [35,36,37,38]. It responds to stressful stimuli and belongs to a danger-associated molecular pattern (PAMP) family. Plants and fungi have closely related proteins, suggesting diverse roles of HMGB1 across the phylogenetic species. HMGB1 acts as an alarmin both extra- and intra-cellularly [39,40,41,42]. Compartmentalization determines HMGB1 function, which is tightly regulated by its molecular binding partners and redox state [43,44,45]. Dendritic polyglycerol sulfates (dPGS) have considerable structural similarities with anticoagulant heparin (sulfated form), which can interact with HMGB1. It is thus anticipated that dendritic polyglycerol sulfates can also bind to HMGB1. The binding of heparin to HMGB1 is mediated via polyion interactions because heparin has negatively charged, and HMGB1 positively charged, domains. Such interactions could occur with negatively charged dendrimers and molecular modeling could predict the dependence of the charge and shape of dendrimers crucial for these polyion interactions. Such interactions are of wide biological interest because they can modulate the cellular locations of HMGB1, thereby modulating its deleterious versus beneficial effects. Considering the ability to tailor the number of charges on the dendrimer surface, they seem to be interesting nano-tools for changing HMGB1 and similar transcription factor locations between the nucleus and cytosol [46]. Tailored polyion interactions between dendrimers and biomolecules will allow for strict control of the number and location of ions on these macromolecules or self-assembled supramolecular structures [46]. Inspiration to study these interactions came from natural interactions, for example, between heparin sulfate and biomolecules [47]. The prevention of IL6 binding to its receptor by dPGS provides protection from excessive cytokine signaling and a vicious circle that propagates inflammation in vivo [31,48]. The concept of dendrimer-mediated modulation of astrocyte state is illustrated in Figure 1. This illustration also shows that another cytokine lipocalin-2 (LCN-2) abundance in astrocytes can be effectively reduced, either by dPGS, redox-regulating N-acetyl cysteine or blocker of toll-like receptors (TLRs), which turn on transcription factor NFκB and propagate inflammation. To study inflammatory processes and the role of microglia in them, several transgenic mouse modes have been used. They are valuable models for in vivo studies of nanostructure impact on neural and other cell types, pharmacokinetics and pharmacodynamics [25]. Such models are particularly suitable for investigations of the mechanisms implicated in microglia regulation upon exposure to different neuroactive agents, toxins and nanostructures including dendrimers. Aside from rodent microglia, human stem cells are valuable sources for microglia, astrocytes, oligodendrocytes, neurons and other cell types [49,50,51,52]. Astrocytes are essential for physiological neuronal functions, but if they acquire genetic mutations, they become highly proliferative and give rise to astrocytoma. Glioblastoma is one of the most malignant tumors, for which there is currently no effective treatment without serious side effects. Examples of dendrimers that have been used in animal models of glioblastoma and other brain tumors are summarized in Table 1. No parallel clinical studies were conducted to date, and only a limited number of reports use cell controls derived from human inducible stem cells. Human astrocytes derived from iPSCs are valuable biological models in monolayer and organoid cultures to systematically investigate anticancer agents incorporated into dendrimer self-assembly systems [25,31,33,34]. The following section briefly describes the microglia and their role under physiological and pathological conditions. It highlights the responses of these glial cells to dendrimers.

### 2.3. Microglia

In the adult brain, microglia are essential surveyors and phagocytes, maintaining homeostasis in the brain [53,54,55,56,57]. Only a limited number of genes and proteins are found in human and rodent glia, and therefore the direct translation of results from animal to human studies is restricted [58,59]. There is no discrete set of biomarkers which uniquely identify microglia under physiological and pathological conditions [60,61]. Among the most commonly used antibodies which recognize microglia and other myeloid linage cells are those binding to C-X3-C Motif Chemokine Receptor 1 (CX3CR1), ionized Calcium-binding Adaptor 1 (IBA1), CD11b, and CD40 [62,63,64,65]. Microglia are extremely dynamic and evidence for this behavior was first recorded in real-time using confocal microscopy [66]. The mobility and chemical mediators they produce allow for an intense cross-talk between astrocytes and other glial cells and neurons [31,48,56,67,68]. Dendrimers with intrinsic anti-inflammatory activity, or those with incorporated anti-inflammatory drugs, are useful nanotechnological tools to modulate neural cell cross-talks [31]. Examples of different dendrimers with and without anti-inflammatory drugs are given in Table 2.

## 3. Dendrimers

### 3.1. Hyperbranched Macromolecules and Their Structure Modification

In 1978, Vögtle et al. reported the first example of hyperbranched macromolecules, which he classified as “cascade” molecules with spherical shape and monodispersity [69]. Subsequently, such tree-like architectures were termed dendrimers by Tomalia in 1985, and have been extensively studied as emerging nanotechnology in recent years [70]. Dendrimers of 5–20 nm size present interesting properties due to their globular shape, controlled layer-by-layer structure, multiple surface functionalities, and high surface area, which are ideally suited for biomedical applications, including the delivery of therapeutics and other bioactive agents, gene transfection, diagnostics, imaging tools and biosensors.

**Figure 2:**
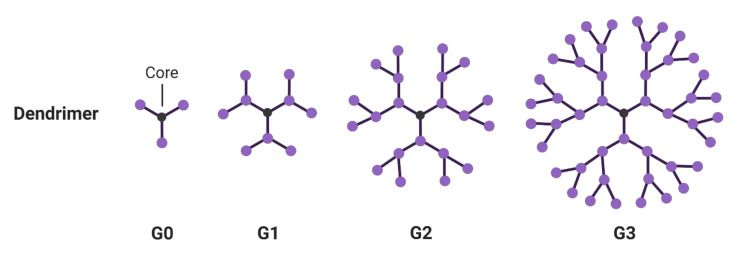
Structure of dendrimers at different generations.

Dendrimers can be constructed by divergent and convergent methodologies, pioneered by Tomalia and Frechét, in which the hyperbranched structure is either built from the core, in a layer-by-layer fashion, or through attaching dendrons to a central core [70,71]. Some well-known dendrimers include poly(amidoamine) (PAMAM), poly(propylene imine) (PPI), phosphorus, and bis-MPA-based dendrimers [72,73,74,75]. Dendrimers are classified by their generation, which is defined as the number of branched layers from a central core, such as G0, G1, G2 (Figure 2). The molecular density of each layer increases logarithmically towards the dendrimer surface. The highest generation number reported to date was a phosphorus-based dendrimer at generation 12 [76]. Kannan et al. showed that dendrimers at a higher generation could perform enhanced accumulation, specificity and retention in the brain tumor model [77]. However, a higher generation number does not always indicate the most efficient encapsulation and therapeutic efficacy. For example, Stenström et al. evaluated the gene transfection efficiency of G2-G4 polyester dendrimers to deliver siRNA in neurons [78]. G2 polyester dendrimers exhibited minimum cytotoxicity, whereas G3 and G4 dendrimers not only displayed concentration-dependent toxicity, but also had compromised transfection efficiency. Interestingly, some low-generation dendrimers without drug payload have been reported to show inherent anti-inflammatory properties, such as G4 PAMAM dendrimers and G3 dPGS [79,80,81].

Click chemistry for dendrimer synthesis has not only contributed to their facile preparation, but also to the addition of a variety of functional units post-synthesis [82]. For example, Neibert et al. showed the facile expansion of the dendrimer core from G0 to hydroxyl-terminated G1 dendrimer through the copper(I)-catalyzed alkyne-azide cycloaddition (CuAAC) click reaction [81]. The internal core structure in high-generation hyperbranched structures, in principle, can enable dendrimers to adapt and encapsulate therapeutic agents [83,84]. However, the major applications of dendrimers have been through tailoring their backbone and surfaces. The latter also plays a significant role in determining their biocompatibility and multitasking ability. PAMAM dendrimers were among the first ones investigated, and since then have been widely studied for their biological applications. These are now available in large quantities from commercial sources and can be modified by simple synthetic procedures. Clinical uses of PAMAM dendrimers have been limited due to their cytotoxicity and rapid clearance, mainly owing to the positively charged amine groups [85]. These terminal groups are then utilized for PEGylation, acetylation, conjugation to carbohydrates, peptides, and other bioactive molecules [86,87]. For instance, PEGylated PAMAM dendrimers have been shown to possess higher drug-loading capacity, enhanced hydrophilicity and biocompatibility, prolonged plasma circulation, improved stability of drug cargos, lowered cytotoxicity and decreased susceptibility to immunogenicity [88,89]. Surface decoration with functional groups allows dendrimers to incorporate drug molecules via covalent interactions, achieving multi-drug delivery and targetability to specific receptors or molecules [90]. By recruiting corresponding ligands or stimuli-responsive units on the surface, dendrimers have been extensively developed as potent nano delivery systems for targeted therapies, particularly for cancer treatments.

### 3.2. Dendrimers to Deliver Agents to Brain Tumors

**Figure 3:**
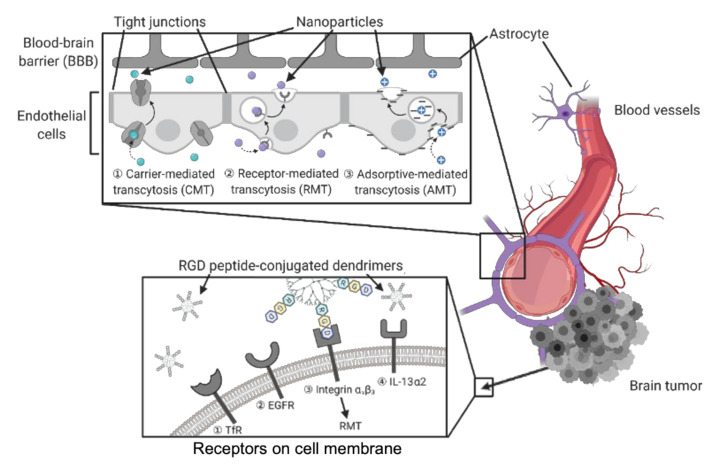
Modes of nanostructures entry through blood–brain barrier (BBB) or brain tumor cells.

As one of the most aggressive and lethal types of cancer, brain tumors are often associated with poor prognosis, strong metastatic ability and high risk of recurrence [91,92]. For example, glioblastoma is a common type of malignant brain tumor derived from astrocytes (astrocytomas) and is widely used as a biological model for studying brain cancer [93,94,95]. Due to a complicated tumor microenvironment (TME) and heterogeneity, current cancer treatments in clinics always require combination therapy, including surgery, chemotherapy and radiotherapy [96,97,98]. Regarding the surgical complications in the brain area, there have been tremendous studies in developing drug delivery systems through non-invasive administration routes. Nevertheless, the blood–brain barrier (BBB), a highly regulated and selective barrier constructed by endothelial cells, prevents efficient drug penetration into the central nervous system (CNS) upon systemic administration [99]. Therefore, BBB-penetrating drug vehicles are necessary to deliver therapeutic agents to brain tumor sites for both chemotherapy and diagnostic imaging. Some routes of administration (e.g. intranasal and invasive intracranial) can bypass BBB and deliver agents directly into the CNS. The cytotoxicity of the delivered agents to healthy cells also needs to be evaluated and minimized [100]. Both endothelial cells and glioma cells express abundant receptors on their surface membrane, which facilitate transcytosis or endocytosis pathways. These receptors can be selectively targeted by the nano delivery systems conjugated to corresponding ligands, such as peptides, folate and carbohydrates [72]. Hence, different modes of BBB penetration and tumor cell uptake can be achieved by such targeted nanoparticles for drug delivery (Figure 3).

To avoid possible brain injury from transient disruption of BBB, and in an effort to enhance permeability, many alternative approaches have been developed. These non-invasive transport pathways include carrier-mediated transcytosis (CMT), receptor-mediated transcytosis (RMT), adsorptive-mediated transcytosis (AMT) [99]. For instance, transferrin (Tf) receptors are highly expressed on the endothelial cell membrane of BBB and mediate iron transport in CNS. BBB targeting transport through Tf receptor (TfR) pathway has been suggested to undergo two alternative mechanisms, RMT and receptor-mediated endocytosis. Kuang et al. conjugated the Tf mimics, peptide T7 (His-Ala-Ile-Tyr-Pro-Arg-His), to PEG-based dendrimers as gene delivery vehicles for interference RNA (RNAi) therapy [101]. Therapeutic genes, such as small interfering RNA (siRNA) and DNA plasmids (pDNA), are easily degraded, whilst nanocarriers like dendrimers can stabilize them and facilitate their biodistribution [102,103]. DGL-PEG-T7/DNA NPs showed extraordinary gene silencing activity and dual-targetability to BBB as well as glioma cells expressing TfRs. Unlike such TfR-targeted nanoparticles, the PAMAM dendrimers synthesized by Liu et al. simultaneously recruited two different ligands, angiopep-2 (Ang2) and epidermal growth factor receptor (EGFR)-targeting peptide (EP-1), to achieve the dual-targetability [104]. Ang-2 peptides are specific ligands binding with lipoprotein receptor-relative protein-1 (LRP1) on endothelial cells of BBB. EP-1 peptides were screened for targeting EGFRs overexpressed by glioma cells. Hence, the BBB penetrability and tumor-targeting efficiency of the dendrimers were enhanced by LRP1-mediated transcytosis and EGFR-mediated endocytosis, respectively. Without conjugation to a ligand specific to a membrane receptor, Muniswamy et al. synthesized PAMAM dendrimers decorated with cationic albumin on surface, thus promoting the penetration of nanoparticles into BBB through AMT and electrostatic interaction [105].

Similar to the principle of nanoparticle entry through BBB, the tumor targetability and cell uptake of nanodelivery systems are accomplished by ligand–receptor interactions. Uram et al. employed biotinylated PAMAM G3 dendrimers and studied the synergistic effects by the loaded cycolxygenase-2 (COX-2) inhibitor, celecoxib, and the peroxisome proliferator-activated receptor γ (PPAR γ) agonist, Fmoc-L-leucine [106,107]. The nanoparticles obtained cell type-dependent cytotoxicity owing to the varied basal levels of biotin receptors, COX-2 and PPAR γ upon biotin-specific uptake by tumor cells and healthy controls. Integrin αvβ3, a cell adhesion molecule overexpressed by many types of tumor cells, is involved in cancer progression and targeted by nanostructures for tumor cell uptake through integrin-mediated endocytosis. The peptide motif Arg-Gly-Asp (RGD) was identified to selectively interact with integrin αvβ3 with high affinity. Zhang et al. designed RGD-modified PEGylated PAMAM dendrimer with acid-sensitive cis-aconityl linkage (RGD-PPCD), which resulted in controlled drug release in weakly acidic TME [108]. In an orthotopic murine model of C6 glioma, doxorubicin (DOX)-conjugated RGD-PPCD showed promoted tumor accumulation and prolonged half-life compared to the free DOX. Even though RGD-PPCD exhibited lower tumor accumulation compared to controls without acid-sensitive bonds, the results suggested efficient RGD-targeting accumulation and RGD-PPCD-treated animal models showed the longest survival times. As listed and explained in Table 1, interleukin-13 receptor α2 and fibrin are also common targets for drug delivery to brain tumors due to their abundance on glioma cells [109,110]. Herein, dendrimers could be directed to the desired brain tumor sites by the corresponding peptides conjugated to them, and then undergoing cell internalization mediated by targeting receptors. Nevertheless, such peptides conjugated to the dendrimer surface are susceptible to proteolysis, followed by a loss of efficiency and off-target effects. Therefore, more in vivo studies are needed to evaluate the stability of such nanoparticles.

**Table 1:** Strategies for dendrimers to penetrate blood–brain barrier and target brain tumors.

Drug or Genetic Cargo	Ligand Modification and Targeted Receptors	Other Main Features	Reference
**Poly**(**amidoamine**) (**PAMAM**)**-Based Dendrimers**			
Doxorubicin	Cationic bovine serum albumin targets negatively charged endothelial cell membranes.	Protonation of free amine groups on dendrimer surface in the acidic environment of tumor tissue.	[105]
Nimesulide	-	PAMAM G3 dendrimer, modified with glycidol, and mixed with G0 PAMAM, reduces systemic cytotoxicity.	[111]
Apoptin	-	Short peptide chains on dendrimers hydrolyzed by peptidase, facilitate the release of positively charged ions and molecules, disrupt the membrane, resulting in endosomal escape via proton sponge effect.	[112]
Celecoxib, Fmoc-L-leucine	Biotin targets cancer cells overexpressing biotin receptors.	-	[106,107]
-	Intrinsic targeting ability to activated microglia/macrophages in CNS by hydroxyl-terminated G4 dendrimers.	-	[113]
Epirubicin, Let-7 miRNA	-	Positively charged surface with Gd and nanographene oxide used for loading drugs through adsorption and electrostatic interactions for combination therapy.	[114]
microRNA 21 (miR-21) inhibitor	-	miR-21 inhibitor loaded dendrimers enhance chemosensitivity of glioblastoma cells to paclitaxel through EGFR/STAT3 signaling.	[115]
**Polyethylene Glycol** (**PEG**)-**Based Dendrimers**			
Bortezomib	Cyclo (Arg-Gly-Asp-D-Tyr-Lys) peptide selectively binds the integrin α_v_β_3_ on cell membrane, resulting in integrin-mediated endocytosis.	Sustained drug release by weakening conjugation between bortezomib and dopamine upon acidic stimuli.	[116]
Quercetin, acetazolamide, lipoic acid	-	Telodendeimer micelles with covalently linked and physically entrapped drugs for combination therapy. Loading efficiency dependent on the physical fit between the drug and micelle core structure.	[83]
pDNA, RNAi	Peptide T7 (His-Ala-Ile-Tyr-Pro-Arg-His) specifically targets brain endothelial and cancer cells overexpressing transferrin (Tf) receptors.	-	[101]
**PEGylated PAMAM-Based Dendrimers**			
Doxorubicin	Angiopep-2 binds low-density lipoprotein receptor-relative protein-1 (LRP1) on the endothelial cells of BBB. EP-1 peptide screened to target epidermal growth factor receptors (EGFRs).	-	[104]
Mesenchymal-epithelial transition (MET)-targeting cMBP peptide	Aberrant MET activation targeted which normally associates with invasiveness and drug resistance of gliomas.	-	[117]
Cytotoxic peptide KLAK	Dissociation of the matrix metalloproteinase 2 (MMP2)-sensitive peptide triggers PEG deshielding, and leads to exposure of the cell-penetrating peptide.	-	[118]
-	Glioma homing peptides (Pep-1) specifically bind the overexpressed interleukin-13 receptors α2 (IL-13Rα2) on glioma cells.	-	[109]
Doxorubicin	Tripeptide Arg-Gly-Asp (RGD) can identify and bind the integrin α_v_β_3_ on cell membrane.	-	[108]
**Dendritic Polyglycerols** (**dPGS**)			
Paclitaxel	Neural cell adhesion molecule (NCAM) overexpression has been found in many tumor cells and correlates with metastasis.	Dendrimer conjugated with NCAM-targeted peptide (NTP) efficiently inhibits endothelial cell migration and offers anti-angiogenesis potential.	[119]

Nearly all the dendrimer-based nanocarrier examples mentioned above share some common properties which render them as favored drug delivery conjugates, compared to free drug administration [120,121]. Generally, nanocarrier shells provide higher hydrophilicity and enhanced stability to the encapsulated drug payloads, preventing non-specific cytotoxicity to healthy tissues and undesired metabolic breakdown of the delivered agents [122]. Moreover, the pharmacokinetic profiles of delivered drugs will be altered upon the structure or surface modifications in nanocarriers, prolonged circulation time, promoted accumulation and penetration at the targeting tissue, as well as higher resistance to degradation and cellular efflux [123]. More importantly, nanostructures are able to offer platforms for multi-drug delivery, thus giving rise to the possibility of combination therapies for better therapeutic efficacy [124,125,126].

To achieve combination therapy, Choi et al. adopted telodendrimers, which are linear-dendritic copolymer hybrid systems for tailored drug delivery [83]. Telodendrimers were composed of linear polymeric blocks and hyperbranched dendrons, giving rise to both the self-assembling property and surface modification capability [127]. Unlike many common dendrimers that solely incorporate drug cargos on their hyperbranched surface, telodendrimer micelles can encapsulate drugs into their hydrophobic cores, while another therapeutic agent can be covalently attached to the surface of the dendron. By synthesizing telodendrimers from tripropargylated pentaerythritol core-based bis-MPA dendrons and linear PEG chains, Choi et al. covalently linked lipoic acid at the dendron surface and physically entrapped quercetin or acetazolamide in the micelles. Telodendrimer micelles conjugated with lipoic acid and physically entrapped quercetin did not exhibit a favored antioxidant effect in LPS-activated microglia compared to free drugs and drugs loaded in PEG-PLGA micelles. In contrast, the cytotoxicity of acetazolamide-incorporated telodendrimer micelles was significantly enhanced compared to controls in either the spheroids or monolayers of U251 glioblastoma cells. Such differences suggest that the encapsulation efficiency and therapeutic effect of drug-loaded telodendrimer micelles depend on a physical fit between the drug cargo and micelle core. In addition to quercetin in this study, many clinical and preclinical studies were carried out with natural products like curcumin, resveratrol and fisetin. All of them stimulated the development of different nanodelivery systems, some of which are based on dendrimers [128].

Yang et al. have developed multi-tasking nanoparticles for simultaneous delivery of the anticancer drug epirubicin (EPI) and Let-7g miRNA based on G4 PAMAM dendrimers, which were modified with gadolinium and nanoscale graphene oxide (Gd-NGO) [114]. As a tumor0suppressor intensively studied for gene-targeting therapy, Let-7g miRNAs decrease expression of the Ras oncogene family, which is normally upregulated in many cancer cells. The overexpression of Let-7g miRNA selectively inhibits proliferation and migration in glioblastoma cell culture but not in normal astrocytes, suggesting that Let-7 miRNA delivery could be a promising therapy for glioblastomas [129]. In order to achieve simultaneous delivery, Yang et al. employed polyethylene glycol (PEG)-functionalized NGO, a nanomaterial that was studied for its potential to deliver either oligonucleotides or anti-cancer drugs, such as doxorubicin and epirubicin [114]. PEG-NGO modified dendrimers were able to load epirubicin non-covalently and adsorb Let-7g miRNA through electrostatic interaction. NGO can not only protect the delivered oligonucleotides from intracellular degradation by enzymes, but also load drugs efficiently and achieve a controlled release that is dependent on pH. During the first 18 h, only 15% of EPI was released under a neutral condition (pH = 7.4) and no further release was observed. However, the release of EPI was accelerated at pH = 6.0 owing to the protonation of amine groups of EPI. In contrast, the release of Let-7g was markedly delayed, as Let-7g was shielded by outer layers from the surrounding medium, suggesting selective release of Let-7g in response to acidic tumor microenvironments. The conjugate was further functionalized with gadolinium, so the resultant Gd-NGO/Let-7g/EPI can be detected by magnetic resonance imaging, identifying tumor and BBB opening sites, and quantifying the delivered agents.

In addition to the emerging trend of developing nano delivery systems integrating ligands specific to receptors expressed on the tumor cell membrane, tumor targetability can also be achieved by “smart” formulations using tumor microenvironment (TME) factors, such as pH, reactive oxygen species, glutathione and enzymes [130]. For example, aberrant mesenchymal–epithelial transition (MET) activation and overexpressed neural cell adhesion molecule (NCAM) are two targetable factors that have been normally found in the TME of gliomas [117,119]. Moreover, the presence of these correlates with tumor cell invasion, drug resistance and angiogenesis. Therefore, nanocarriers conjugated with homing peptides to such TME factors are more likely to accumulate in the growing tumor area, such as tumor cells undergoing angiogenesis. However, the NCAM-targeting PEGylated dPGS conjugate developed by Vossen et al. only accumulated in human umbilical vein endothelial cells (HUVEC) in vitro, which had NCAM during angiogenic process. The enhanced therapeutic effect of the drug payloads was not shown in in vivo experiments using a murine model of neuroblastoma. As we discussed before, although the encapsulated drugs, or therapeutic peptides, could be spared from degradation owing to protection by the nanoparticle shell, tumor-homing peptides decorated on the nanocarrier surface are at high risk of being cleaved by proteases in vivo.

As another widely studied TME target for cancer diagnostics, matrix metalloproteinase (MMP) enzymes have been found to be overexpressed in many types of tumor sites. Han et al. reported the first tumor microenvironment-responsive polysaccharide-decorated dendrimer developed for size-shrinkable drug delivery by attaching MMP-2 cleavable peptides (PLGLAG) [131]. Hyaluronic acid (HA) terminal groups were attached to PAMAM core through PLGLAG peptide linkages, which can be cleaved by MMP-2 at tumor sites, and the dendrimer will shrink from ~200 to ~10 nm. The size shrinkage property benefits the cleaved dendrimers in the enhanced permeability and retention (EPR) effect, thus improving the cellular uptake of nanoparticles [132,133]. Liu et al. also employed MMP2-sensitive peptide (GPLGIAGQ) in generation 4 PAMAM dendrimers for delivering cytotoxic peptide KLAK [118]. Unlike previous examples showing potential drawbacks in peptide-conjugated nanocarriers, Liu et al. demonstrated the opposite proteolytic properties. The cleavage of an MMP2-sensitive peptide leads to the dissociation of PEG corona and exposure of KLAK and transactivating transcriptional activator peptide (TAT), a cell-penetrating peptide. The outer PEG shell improves the biocompatibility of the dendrimer and prevents nonspecific cytotoxicity of KLAK, maintaining the stability of the nanoparticles. The diameter of PEG-shielded dendrimers remained constant for 24 h, whereas the size of the unprotected dendrimers only lasted for 4 h. Following deprotection by the removal of PEG, significantly deeper penetration of the dendrimers, either mediated by TAT or through endocytosis, was observed in U87 glioblastoma spheroids. As KLAK destroys the mitochondrial membrane, thus inducing a cell apoptosis cascade, the anticancer activity of dendrimers was then confirmed by JC-1 assay. Interestingly, the cytotoxic effect of dendrimers was enhanced in U87 cells which expressed higher levels of MMP compared to MCF-7 cells with a milder expression of MMP, even at low concentrations of dendrimers. This indicates a promising cancer-cell-selective peptide delivery in deep-seated solid tumor therapies.

### 3.3. Dendrimers to Deliver Anti-Inflammatory Agents

Whilst dendrimer-based drug formulations for brain tumors have faced several challenges, there are already commercially available polymer-drug nanoconjugates for anti-infectious and anti-inflammatory treatments. For example, as one of the most successful products of nanomedicine, liposomal formulation amphotericin B (AmBisome^®^) has provided a superior pharmacokinetic profile, while retaining antifungal bioactivity [134]. Meanwhile, Starpharma successfully made progress in designing the polylysine-based dendrimer, SPL7013, and developed it as a gel-formulated vaginal microbicide (VivaGel^®^) to prevent HIV and HPV infection [135,136]. However, expensive costs in synthesis, and difficulties in reproducibility and scaling-up have usually limited the translation of nano delivery formulations from lab work to industrial production. Notably, there are many dendrimer drugs in preclinical studies or in clinical trials developed for the treatments of rheumatoid arthritis, retinal degeneration, tuberculosis and lung inflammation [137,138,139,140,141]. The translation of dendrimer-based drugs from in vitro experiments to clinical use is still in early stages. Dendrimers have offered a promising therapeutic property, which is the inherent anti-inflammatory activity of the naked dendrimer structures without any drug payload. This was first reported using PAMAM-based dendrimers by Tomalia et al. using three different animal models of inflammation [79]. Surfaces of generation 4 or 4.5 PAMAM dendrimers were terminated with amine groups, hydroxyl groups and carboxyl groups, respectively. All three types of PAMAM dendrimer exhibited anti-inflammatory effects in the carrageenan-induced paw edema model, while G4-NH_2_ and G4-OH showed a higher mean percent inhibition overall. This exciting result indicates potential strategies employing certain surface-functionalized PAMAM dendrimers for anti-inflammatory therapies.

PAMAM dendrimer-based drug delivery of anti-inflammatory agents has also been studied in a wide range of biological models. The most well-studied nano delivery system is PAMAM-G4-OH dendrimer, which was tested for treatments of many inflammation-associated diseases, such as trauma, cerebral palsy, retinal degeneration and inflammation-induced preterm birth [139,142,143]. PAMAM-G4-OH can inherently target activated microglia and macrophages (Mi/Ma) at inflammatory regions and shows outstanding compatibility with different therapeutic drugs, including N-acetyl-L-cysteine, sinomenine, minocycline, dexamethasone and fluocinolone acetonide [139,142,143,144,145,146]. In a recent report, generation 2 PEG-based dendrimer functionalized with highly dense hydroxyl surface terminals (PEGOL-60) was synthesized, which exhibited superior performance to PAMAM-G4-OH, especially in selective targetability to neuroinflammation [147]. Dendrimers with a PEG backbone benefit from enhanced biocompatibility, aqueous solubility, stability and reduced immunogenicity. Moreover, compared to PAMAM-G4-OH, which requires more than eight steps during synthesis, the synthesis of PEGOL-60 is much less complicated and more efficient. Owing to Cu(I)-catalyzed alkyne azide and thiol-ene click chemistry, the reaction time of PEGOL-60 synthesis is largely shortened from the core extending to the periphery. In in vivo studies, PEGOL-60 is not only able to cross biological barriers including the blood–brain barrier (BBB) and localize at activated Mi/Ma, but also shows intrinsic antioxidant and anti-inflammatory behaviors. The high tumor penetrability, inherent targetability and bioactivity of PEGOL-60 suggest the potential for a potent targeted systemic drug delivery approach for anti-inflammatory therapies in CNS.

Dendritic polyglycerol sulfate (dPGS) is a hyperbranched polyglycerol scaffold functionalized with peripheral sulfate groups, and its intrinsic anti-inflammatory activity has been extensively studied. Dernedde et al. first reported the inhibition of cell adhesion molecules leukocytic *L*- and endothelial P-selectin by dPGS, hence preventing leukocyte extravasation and alleviating chronic inflammation in a contact dermatitis mouse model [80]. In addition, dPGS also interacted with complement factors C3 and C5, resulting in reduced anaphylatoxin C5a level in vivo. As anionic sulfate groups of dPGS are supposed to target and bind selectins at positively charged protein motifs, selectin binding affinity is determined by the size and degree of sulfation of dPGS.

**Figure 4:**
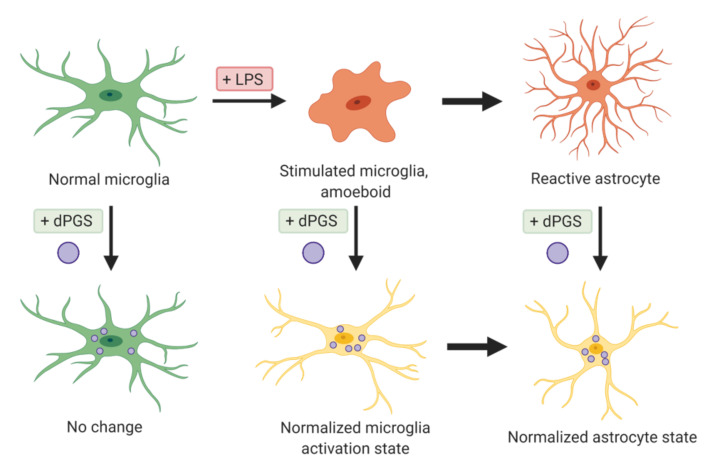
dPGS attenuate microglia activation and reactivity of astrocytes.

In recent years, Maysinger’s group has studied the role of dPGS in the modulation of neuroinflammation, and their effects especially on glial cells (Figure 4). dPGS significantly reduced the generation of proinflammatory cytokines and nitrites from lipopolysaccharide (LPS)-activated microglia, suggesting prevention of the deleterious transformation of microglia phenotype in response to inflammatory stimuli [32]. dPGS also normalized the LPS-induced morphology of hippocampal dendritic spines and restored the function of CA1 pyramidal neurons. In established Alzheimer’s disease models exposed to neurotoxic 42 amino acid amyloid-β (Aβ42) peptide, dPGS was found to prevent Aβ fibril formation through direct binding after internalized by glia cells and hippocampal slice cultures [22]. dPGS not only prevented Aβ peptide accumulation in neuroglia and loss of excitatory postsynaptic dendritic spines, but also attenuated Aβ42-induced glial hyperactivation by reducing lipocalin-2 (LCN-2) production mainly in astrocytes. The overall results suggest a potential use of dPGS in inflammation-associated neurological disorders. Some representative dendrimers with intrinsic anti-inflammatory properties and delivering anti-inflammatory agents are summarized below (Table 2 and Table 3).

**Table 2:** Dendrimers with intrinsic anti-inflammatory activity.

Modification	Disease Models	Other Main Features	Reference
**Poly**(**amidoamine**) (**PAMAM**)-**BASED Dendrimers**			
Surface-modified anionic G4.5-COOH and neutral G5-OH.	Mouse model of acute pancreatitis	Inhibition of macrophage infiltration and suppression of pro-inflammatory cytokine expression.	[148]
PAMAM or poly(ethylenimine) dendrimers immobilized onto PSMA/polystyrene microfiber meshes, generating nucleic acid-binding polymers.	Human cancer and mouse macrophage cell lines	Inhibition of DAMP-mediated TLR stimulation and thrombosis by scavenging exDNA and HMGB1. Attenuation of inflammatory responses and coagulation induced by traumatic injury.	[149]
Simple surface modification of PAMAM dendrimers with -NH_2_, -OH, and -COOH.	Rat models of inflammation	The first study on intrinsic anti-inflammatory activity of PAMAM dendrimers.	[79]
**Polyethylene Glycol** (**PEG**)-**Based Dendrimers**			
Highly dense surface hydroxyl terminals.	Models: Rabbit, cerebral palsy; Murine, glioblastoma; rat, age-related macular degeneration	Ability to cross CNS barriers, including BBB, blood–retinal barrier (BRB), and blood–brain-tumor barrier (BBTB). Selectively targets activated microglia/macrophages in CNS in vivo upon systemic administration. Intrinsic anti-oxidant and anti-inflammatory activities in vitro.	[147]
**Polyglycerol-Based Dendrimers**			
Dendritic polyglycerols were either terminated with hydroxyl groups (dPG) or sulfate groups (dPGS).	Mouse primary cortical cultures; mouse model of microglial cell activation	dPGS alleviated LPS-induced microglia activation, reduction in LCN-2 production mainly in astrocytes. dPGS directly bound to IL-6 and LCN-2, attenuating astrocyte stimulation.	[31]
G3.5 dPGS	Organotypic hippocampal slice cultures	dPGS treatment in Alzheimer disease models prevents Aβ fibril formation by directly interacting with the Aβ_42_ peptide, and attenuating Aβ-induced neuroinflammation.	[22]
Sulfated polyglycerols (dPGS) and non-sulfated analogs (dPG).	Organotypic hippocampal slice cultures	dPGS reduces pro-inflammatory cytokine production from M1 microglia phenotype, and normalizes LPS-induced morphology of the hippocampal dendritic spines.	[32]
Anionic dPGS moieties interact with the ligand binding sites of P- and L-selectin through electrostatic interactions.	Mouse model of contact dermatitis and complement activation	The first report about the anti-inflammatory activity of dPGS.	[80]
**Phosphorus-Based Dendrimers**			
Fluorescent phosphorus dendrimers.	Murine macrophages (M1 and M2 phenotypes)	Generation of the phenotype-dependent blue spectral shift upon macrophage polarization, potential use of biosensor identifying macrophages and their phenotypes.	[150]
**Polyphosphorhydrazone** (**PPH**)**-Based Dendrimers**			
Azabisphosphonated (ABP) surface modification imparts anti-inflammatory activity to the dendrimer.	Mouse model of experimental autoimmune encephalomyelitis and arthritis. Human peripheral blood mononuclear cell line.	Attenuation of the pathological symptoms and mediation of the inflammatory response through regulating immune cells and decreased cytokine release.	[137,138,151]

**Table 3:** Dendrimers to deliver anti-inflammatory agents.

Drug or Genetic Cargos	Modification	In Vitro or In Vivo	Reference
Nitric oxide (NO)	Dendrimer surface modified with 18 NO-releasing moieties.	In vitro	[152]
*N*-Acetyl-*L*-cysteine (NAC)	Triphenyl-phosphonium ligand modification enables mitochondrial targeting delivery of NAC.	In vitro and in vivo	[142,144]
-	Surface decoration with carbohydrate-based targeting moieties contributes to the macrophage-targeting ability of nanoparticles.	In vitro	[153]
-	Increased cellular uptake of mannose-conjugated dendrimers preferentially by injured microglia through mannose receptor-mediated endocytosis.	In vitro and in vivo	[154]
*N*-acetyl-*L*-cysteine (NAC)	The penetration enhancer Capmul MCM (glycerol monocaprylate) benefited in designing oral formulations of NAC.	In vitro and in vivo	[155]
Dexamethasone	Hyaluronic acid-conjugated dendrimers were synthesized as a subconjunctival injectable gel.	In vitro and in vivo	[146]
*N*-acetyl-*L*-cysteine	Positive therapeutic effects in the fetus and the newborn upon intra-amniotic administration.	In vivo	[143]
Triamcinolone acetonide	Inhibition of nerve injury-induced microglial activation and reduced neuropathic pain upon intrathecal administration.	In vitro and in vivo	[156]
-	Intravenous or intravitreally administered dendrimers could be a safer drug delivery approach compared to the current therapy, which requires direct injection in the eye.	In vivo	[157]
**PEGylated PAMAM-Based Dendrimers**			
Scutellarin	Dual targetability owing to angiopepsin-2 and *N*-acetylated proline-glycine-proline (PGP), which selectively bind with LRP in BBB endothelial cells and CXCR2 in infiltrating neutrophils respectively.	In vitro and in vivo	[158]
-	Folate-conjugated dendrimers target the folate-receptor positive macrophages which play a significant role in mediating inflammatory response.	In vitro and in vivo	[159]
**Phosphorus-Based Dendrimers**			
TNF-α siRNA	The cationic phosphorus dendrimers were modified with either pyrrolidinium or morpholinium terminal groups in order to improve biocompatibility of dendrimers and complexation with siRNA.	In vitro and in vivo	[141]

### 3.4. Applications of Dendrimers Aside from the Delivery of Therapeutic Agents

Dendrimers have also been developed as probes for resolving issues with fast leakage of fluorescent probes. By adopting PAMAM-based dendrimers loaded with small fluorescent dyes, Lamy et al. developed a nanoprobe specialized for sodium sensing in neurons with high stability and sensitivity [160]. Most studies of ion imaging have focused on calcium signaling, since the large amplitude of Ca^2+^ signals enables easy detection compared to other ions, such as Na^+^ ions. To optimize the loading of Na^+^-sensitive dyes, CoroNa Green (CG) and CoroNa Red (CR), generation 4.5 or 5 PAMAM dendrimers with three types of surface functionalization were tested, including positively charged amine groups, neutral PEG chains and negatively charged carboxyl groups. NH_2_-modified dendrimers had a better binding capacity for anionic CG molecules than PEGylated dendrimers, suggesting different complexions between dyes and dendrimers. Dendrimer-based nanoprobes not only encapsulate small molecule dyes without complicated covalent chemistry, but also prolong the intracellular half-life of loaded dyes with better stability [161,162]. Moreover, the large size of fluorescent dye-incorporated dendrimers allows nanoprobes to be spared from rapid cellular efflux, resulting in improved intracellular retention and more accurate measurement of dynamic changes [163]. This dendrimer-based nanoprobe is straightforward to synthesize, performs enhanced ion imaging, and provides a versatile platform for many other fluorescent dyes.

**Figure 5:**
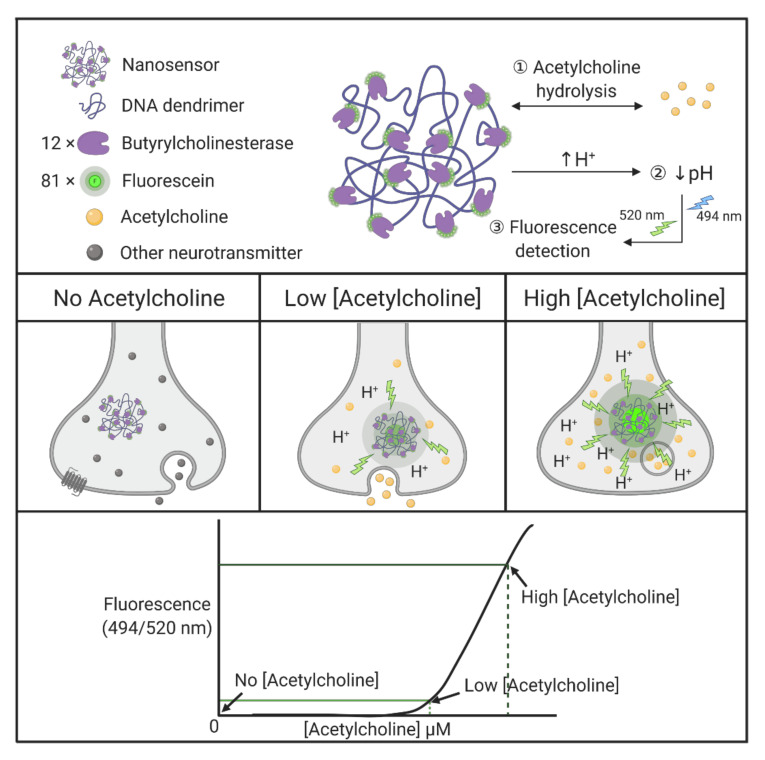
Enzyme-linked acetylcholine-responsive nanosensor.

It is rather demanding to measure the dynamics of small molecule movements within living cells. The ability to record and measure spatial and temporal changes in single cells, by monitoring discrete changes in, for example, neurotransmitters within a synapse, is important for better understanding molecular and cellular mechanisms in neural cells. Walsh et al. developed nanosensors specific to the detection of synaptic acetylcholine, based on DNA dendrimer scaffolds modified with enzymes and fluorophores [164]. Butyrylcholinesterase, an enzyme that selectively hydrolyzes acetylcholine, is covalently conjugated to the DNA dendrimer, together with fluorescein. Followed by lowering of local pH upon acetylcholine hydrolysis, pH-dependent fluorophore molecules are able to generate highly sensitive signals, despite likely photobleaching and degradation. To optimize the nanosensor response, 81 fluorescein molecules and 12 butyrylcholinesterase molecules are evenly spaced and attached to the DNA backbone (Figure 5) [164]. The resultant dendrimer-based nanosensors have diameters of 130 ± 24 nm. The nanoscaled sensors take advantage of processing single synapses, whereas typical electrode techniques are limited by their sizes. More importantly, nonspecific pH fluctuations do not trigger irreversible nanosensor degradation and such modular nanosensors are also capable of adapting other enzyme incorporations. Several other interesting dendrimer applications are summarized in Table 4.

## 4. Conclusions and Future Outlook

We have briefly discussed issues in nanomedicine, focusing mainly on dendrimers and their effects on the transformed (glioblastoma) and untransformed cells (astrocytes and microglia) in the central nervous system. Some key structural, functional and communication characteristics at the nanoscale between neural cells are highlighted. Examples of the use and effects of dendrimers carrying drugs or performing as nanostructures with intrinsic anti-inflammatory properties are presented. Major limitations applying dendrimers in clinical trials for diagnostic and therapeutic purposes in neurological disorders do not appear as much on the dendrimer side, but rather in the complex and still-limited knowledge of the central nervous system fine structures and communications. The neural cell interactions are not only complex but also dynamic and targeting what and when is an issue to be resolved in the future. Dendrimers are perhaps among the most promising nanostructures for tackling the central nervous system impairments due to their defined structural features and elegant chemistry that allows their functionalization. The main message here is that both neurons and glial cells can be affected by nanostructures, and that they communicate between one another utilizing biological nanostructures. Dendrimers and some other nanostructures can exert modulatory effects in glia, neurons and vascular cells in the brain. These modulatory effects are seen at the morphological, signaling and functional levels. The figures and tables provided here summarize some concepts and uses of dendrimers in experimental and clinical medicine.

Where do we go from here? Dendrimers remain one of the most attractive nanostructures for future investigations for their diagnostic and therapeutic purposes. The versatility of their synthesis will expand their utility in nanooncology and nanomedicine. Considering that many biological processes involve polyion interactions and that the number of ions can be easily tailored in dendrimer structures, they should be regarded as high-priority nanostructures in future investigations at multiple levels: (i) synthesis and characterization of stimulus-responsive dendrimers with high specificity, (ii) preparation and application of intrinsically fluorescent dendrimers that can be imaged in living cells and eventually in tissues and (iii) application of dendrimers with small, non-protein targeting moieties to achieve their distribution and cargo delivery in a cell-type and tissue-dependent manner. In this vein, the modulation of organelles in individual cells and tissues with dendrimers, both under physiological and pathological conditions, merits further investigation.

## Figures and Tables

**Table 4:** Representative dendrimers in biomedical applications.

Dendrimer	Application	Main Features	Reference
**PAMAM**	Diagnostic probe; Anti-cancer and antimetastatic agent	PAMAM dendrimers labeled with radioactive 131I and modified with LyP-1 peptides, allow conjugates to target tumors. Used in SPECT imaging, radionuclide therapy, and as an antimetastatic agent.	[165]
**PAMAM**	Drug delivery	Hyaluronic acid terminated surface deshielded through MMP-2 cleavable linkages at tumor sites. Dendrimer size shrinks from ~200 to ~10 nm, promoting EPR effect, facilitating cellular uptake.	[131]
**PAMAM**	Gene transfection	Aptamer S6 against A549 lung carcinoma screened and selected by cell-SELEX.	[166]
**PAMAM**	Nanoprobe	Highly sensitive to dynamic cellular sodium changes, and encapsulates fluorescent dyes.	[160]
**PEGylated PAMAM**	Nanoprobe	MR/CT dual-mode imaging, Dendrimers encapsulate AuNPs for CT imaging, and chelate with Mn(II) for high contrast abilities. Functionalization with RGD peptides leads to avb3 integrin targeting.	[167]
**PEG PAMAM**	Fibrous bio-catalyst	PAMAM or PEG dendrimers grafted on polyester fabrics activated through plasma treatments, followed by immobilization of glucose oxidase enzyme.	[168]
**dPGS PCL**	Drug delivery	Sheddable dPGS shell selectively binds L-selection. Intrinsic targeting ability to inflammatory and tumor sites. GSH-cleavable disulfide bonds provide controlled drug release. The first study showing that dPGS could be a potential alternative to PEG.	[84]
**Polyglycerol**	Fluorescent probe	Polyglycerol dendrimers were conjugated to a BODIPY core for single-molecule imaging.	[169]
**DNA**	Nanosensor	Butyrylcholinesterase and fluorescein-conjugated with DNA dendrimers, generating dendritic scaffolds, act as selective nanosensors for acetylcholine. Butyrylcholinesterase enzymes selectively hydrolyze acetylcholine and lower local pH, followed by detection of the pH-sensitive fluorescent indicator in a single synapse.	[164]
**Amino acid**	Enzyme model; Drug delivery	Dendritic peptides used as models of natural enzymes. The globular shape mimics protein structure and shows catalytic activity.	[170]
